# Oral anticoagulation therapy in nonagenarian patients with atrial fibrillation: usage patterns, predictors, and in-hospital outcomes from a single-center retrospective study

**DOI:** 10.1186/s12877-026-08098-y

**Published:** 2026-08-08

**Authors:** Zinan Zhao, Ying Dai, Wenxing Peng, Ning Sun, Cheng Wu, Pengfei Jin, Yatong Zhang, Yifan Na, Junpeng Liu

**Affiliations:** 1 Department of Pharmacy, Beijing Hospital, National Center for Gerontology; National Clinical Research Center for Gerontology; The Key Laboratory of Geriatrics of NHC; Institute of Geriatric Medicine, Chinese Academy of Medical Sciences, Beijing, P.R. China; 2 Department of Pharmacy, First Affiliated Hospital of Wenzhou Medical University, Wenzhou, China; 3 Department of Pharmacy, Beijing Anzhen Hospital, Capital Medical University, Beijing, China; 4 Department of Cardiology, Beijing Hospital, National Center for Gerontology; The Key Laboratory of Geriatrics of NHC; Institute of Geriatric Medicine, Chinese Academy of Medical Sciences, Beijing, P.R. China

**Keywords:** Atrial fibrillation, Anticoagulants, Nonagenarian, Direct oral anticoagulants

## Abstract

**Background:**

Atrial fibrillation (AF) is highly prevalent in nonagenarians (≥ 90 years). However, this group is often excluded from clinical trials, leaving limited real-world evidence on oral anticoagulant (OAC) use, safety, and efficacy. This study sought to investigate the usage patterns, predictors, and in-hospital outcomes of OAC therapy in nonagenarian patients with AF to provide real-world evidence to inform clinical decision-making.

**Methods:**

A retrospective single-center analysis was conducted on 290 nonagenarian AF patients (Beijing Hospital, 2018–2024). Patients were grouped as OAC (*n* = 89) or non-OAC (*n* = 201). Primary endpoints: In-hospital stroke/SE (efficacy) and major bleeding (ISTH criteria; safety). Secondary endpoints included in-hospital composite efficacy, net clinical benefit (stroke/SE/major bleeding/all-cause death), and all-cause death. Multivariate logistic regression identified OAC predictors; OAC temporal trends were analyzed.

**Results:**

The overall OAC utilization rate was 30.69% and showed a steady annual increase. Positive predictors of OAC use included a history of ischemic stroke (OR = 2.653) and heart failure (OR = 2.083), while negative predictors included bedridden status (OR = 0.134), and concomitant antiplatelet use (OR = 0.203). DOAC accounted for 82.02% of OAC prescription, and 63.01% of DOAC users were inappropriately underdosed. Compared with the non-OAC group, OAC use was significantly associated with a lower risk of in-hospital stroke/SE (6.74% vs. 15.42%, OR = 0.396, 95% CI = 0.159–0.987, *p* = 0.047) and improved secondary composite efficacy outcomes (OR = 0.491, *p* = 0.024). The OAC group also demonstrated a numerically lower incidence of net clinical benefit outcomes (20.22% vs. 30.85%), though it did not differ significantly between groups (2.25% vs. 0.50%, *p* = 0.215), though total bleeding events were higher in the OAC group (49.44% vs. 36.32%, *p* = 0.037).

**Conclusions:**

Although the overall OAC utilization rate in AF patients aged ≥ 90 years remains relatively low, it has shown a steady annual increase, with DOACs emerging as the predominant choice. OAC therapy is significantly associated with a lower risk of in-hospital stroke and demonstrates favorable net clinical benefit, highlighting the potential value of the judicious use of OAC in this population.

**Clinical trial registration:**

Chinese Clinical Trial Registry, identifier ChiCTR2300076161.

**Supplementary Information:**

The online version contains supplementary material available at https://doi.org/10.1186/s12877-026-08098-y.

## Introduction

Atrial fibrillation (AF) is the most common cardiac arrhythmia [1]. The prevalence of AF increases with age, reaching 15% in the group aged older than 85 years [2]. Older patients are at a higher risk of stroke or systemic embolic events (SEE), as well as more serious bleeding complications, like major and intracranial hemorrhage (ICH) [3].

While therapeutic, management strategies and stroke prevention continue to evolve and improve [4], the complexity of anticoagulant therapy in elderly patients with AF — coupled with their historical exclusion from clinical trials — has resulted in a lack of evidence for clinical medication. Given the aging population, evidence supporting effective anticoagulation regimens for high-risk, very elderly patients is essential [5, 6]. Although the recent 2024 ESC Guidelines strongly advocate for individualized thromboembolic prevention, nonagenarians remain a clinical grey zone due to their systematic exclusion from pivotal trials [7].

The ELDERCARE-AF trial showed that once-daily, very-low-dose edoxaban (15 mg) was superior to placebo in reducing stroke/systemic embolism (SE) in AF patients aged ≥ 80 years, and nonsignificantly increased major bleeding [8]. Nevertheless, insufficient real-world evidence exists regarding the clinical efficacy and safety of anticoagulation strategies—including OAC use, non-use, and different OAC types—in very elderly patients with AF, with a notable paucity of data specifically for those aged 90 years and older. Using inpatient data from a single tertiary hospital in China, the present study aims to comprehensively investigate the usage patterns, predictors, and in-hospital outcomes of different anticoagulant regimen in AF patients aged 90 years and older in recent years, evaluate in-hospital event rates associated with different regimens, and provide real-world evidence to support clinical medication decisions.

## Methods

### Study design and participants

This was a retrospective analysis of 290 patients aged ≥ 90 years with atrial fibrillation (2018–2024). Data were extracted from the hospital information system (HIS) of Beijing hospital (a tertiary general hospital) from January 1, 2018 to December 30, 2024. The database encompassed comprehensive details on admission and discharge, age, sex, drinking status, medications, and laboratory test results of the patients. Oral anticoagulant (OAC) use was defined as the patient’s established pre-admission home medication regimen documented upon hospital admission, capturing ongoing chronic therapy rather than acute in-hospital initiation.

### Patient recruitment criteria

The inclusion criteria were: (1) age ≥ 90 years; (2) patients diagnosed with atrial fibrillation or atrial flutter; (3) patients hospitalized for treatment in the Department of Cardiology or Department of Neurology. For patients with multiple related hospitalizations, all admission records were systematically retained to ensure comprehensive data capture.

Patients were excluded if they: (1) received low-molecular-weight heparin for anticoagulant therapy; (2) had absolute contraindications to anticoagulant therapy (e.g., active uncontrollable major bleeding, severe coagulopathy, or acute extensive intracranial hemorrhage at admission); or (3) had absolute indications for chronic oral anticoagulation other than AF (such as the presence of mechanical prosthetic valves, moderate-to-severe rheumatic mitral stenosis, or recent venous thromboembolism requiring ongoing therapeutic anticoagulation).

Trained personnel abstracted demographic, clinical characteristics and scores, laboratory, and treatment-related data of these participants from the electronic medical record system. This included participants’ age, sex, ward of origin (departments of cardiology and neurology), medical scores (CONUT, CHA_2_DS_2_-VASc and HAS-BLED scores), comorbidities [such as hypertension, diabetes mellitus (DM), hyperlipemia, heart failure, and ischemic stroke], treatment variables (i.e., complete medication list), and laboratory values including serum creatinine and hemoglobin.

Comorbidity was defined as the concurrent diagnosis of three or more chronic diseases in the same individual. While the traditional threshold is often set at two conditions, a threshold of ≥ 3 was explicitly chosen for this nonagenarian cohort to avoid the statistical ceiling effect commonly seen in ultra-elderly populations and to more rigorously identify patients with genuine clinical complexity and higher healthcare needs [9, 10]. We used the control nutritional (CONUT) score to assess the nutritional status of the enrolled patients. According to the CONUT scoring system, a score of ≥ 2 is considered malnutrition [11]. Polypharmacy as the consumption of five or more drugs daily in the same individual [12].

### Definition of outcomes

The primary efficacy end point was the stroke or systemic embolism (stroke/SE), and the primary safety end point was major bleeding, as defined by the International Society on Thrombosis and Haemostasis (ISTH). Importantly, to accurately reflect the in-hospital treatment effect, the primary efficacy endpoint of stroke was strictly defined as incident stroke occurring during the hospitalization period. Patients presenting to the hospital with acute stroke as their primary admission diagnosis were recorded in baseline characteristics but excluded from the incident event count. Major bleeding was defined as acute or sub-acute bleeding with clinical manifestations meeting any of the following: fatal bleeding and/or symptomatic bleeding in key sites or organs (e.g., intracranial, intraspinal, intraocular, retroperitoneal, intra-articular, pericardial, intramuscular with fascial compartment syndrome), a hemoglobin drop of ≥ 2 g/dl within 24 h due to bleeding, or transfusion of 2 or more units of whole blood or packed red blood cells.

Secondary efficacy end points included the composite of stroke, or death from all causes; net clinical benefit (the composite of stroke, major bleeding, or death from any cause); and death from any cause. Secondary safety end points included the composite of major bleeding or clinically relevant nonmajor bleeding; clinically relevant non-major bleeding (CRNMB); minor bleeding; and all bleeding. clinically relevant nonmajor bleeding was acute or sub-acute significant bleeding with clinical manifestations (not meeting major bleeding criteria) and accompanied by at least one of: hospitalization due to bleeding, receipt of medical-related hemostatic treatment (medical or surgical), or adjustment of anticoagulation treatment regimen. Minor bleeding was all acute bleeding events with clinical manifestations not meeting major or clinically relevant non-major bleeding criteria.

### Statistical analysis

Patients were divided into two groups: the group not receiving anticoagulation therapy (non-OAC group) and the group receiving oral anticoagulation therapy (OAC group). Most variables in baseline characteristics exhibited non-normal distributions as determined by Shapiro-Wilk tests. Continuous variables were consequently summarized using median and interquartile range (IQR), while categorical variables were expressed as frequencies and percentages. Non-parametric methods were employed for group comparisons: the Mann-Whitney U test or Kruskal-Wallis test for continuous variables, and the chi-square test (with Yates’ continuity correction) or Fisher’s exact test for categorical variables, as appropriate. We used Cochran-Mantel-Haenszel tests to assess the calendar trends of non-OAC, DOAC and vitamin K antagonist (VKA) utilization.

Statistical analyses were conducted using R (v4.2.0) and SPSS Statistics (v27.0, IBM). To strictly prevent model overfitting and multicollinearity, variables demonstrating *P* < 0.05 in the univariate analysis were carefully screened. After excluding post-baseline outcomes and composite scores (e.g., HAS-BLED) that inherently overlap with specific comorbidities, a parsimonious set of seven fundamental clinical variables was included in the final multivariable logistic regression model using the ‘Enter’ method. This strategy robustly adhered to the events-per-variable (EPV) ≥ 10 principle. Independent predictors of OAC prescription were presented as odds ratios (OR) with 95% confidence intervals (95% CI). A two-tailed p-value < 0.05 was considered statistically significant. To address multiple comparisons, the Bonferroni correction was applied by adjusting the significance threshold to 0.05 divided by the number of comparisons. All reported p-values explicitly indicate whether they are raw or adjusted values.

In addition, we conducted a subgroup analysis of patients using DOACs to evaluate the appropriateness of dosing. Each patient’s prescription was strictly verified against the National Medical Products Administration (NMPA) package inserts (detailed criteria are provided in *Supplementary eTable 1*). Patients were classified into two categories: (1) Appropriately Dosed, which included patients receiving the standard full dose as well as those receiving a justified reduced dose that met the specific NMPA criteria (e.g., advanced age, low body weight, moderate renal impairment, or concomitant use of strong P-gp inhibitors); and (2) Inappropriately Underdosed, defined as patients who were prescribed a reduced dose in the absence of any justified criteria for dose reduction.

*Ethics and Trial registration.* The study protocol complied with Good Clinical Practice standards for drugs and the ethical guidelines specified in the revised Declaration of Helsinki (2013). Beijing Hospital Ethics Committee approved this study (Approval Letter Number: 2023BJYYEC-279-01) and the study was registered at Chinese Clinical Trial Registry (Registration number: ChiCTR2300076161). Data extracted from medical records was retrospected, de-identified and anonymized before analysis; therefore, the requirement for informed consent was waived for this study.

## Results

### Participant characteristics

**Fig. 1:**
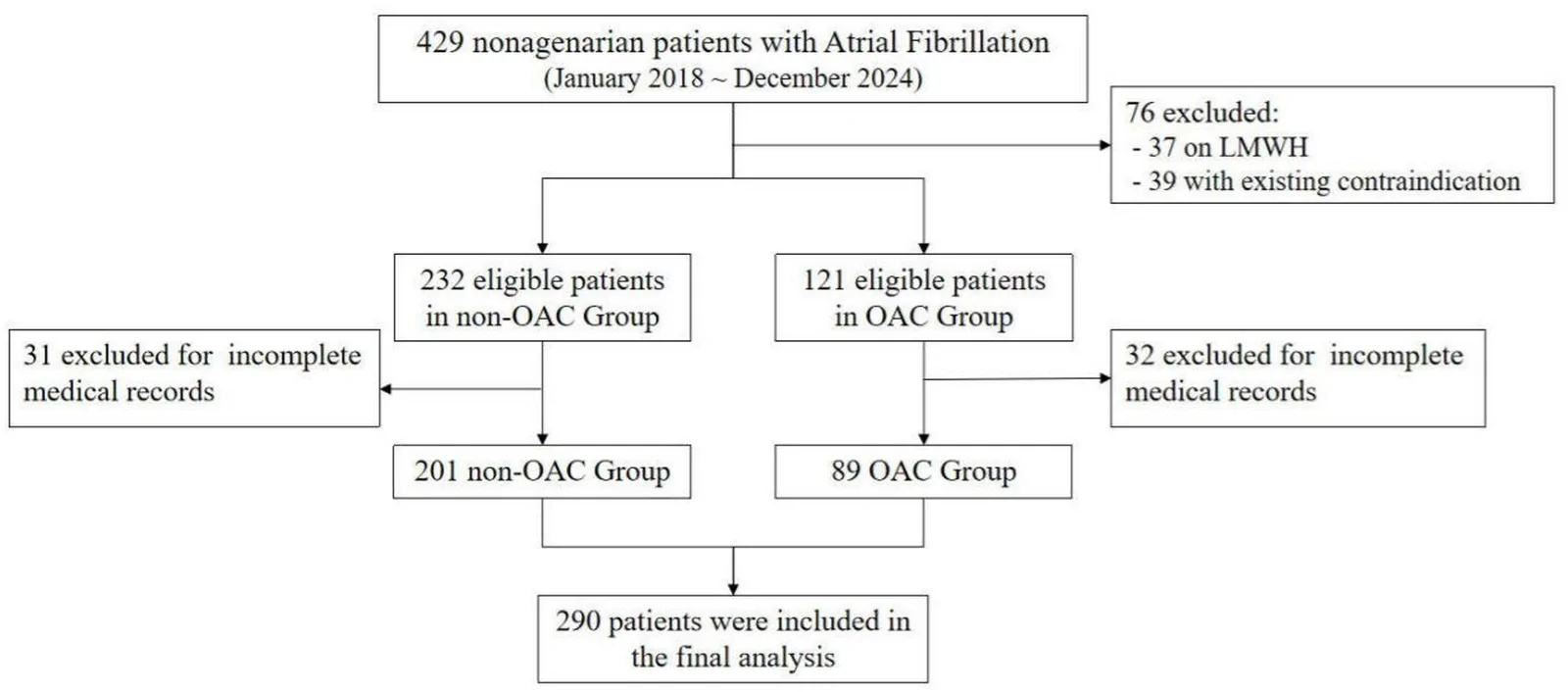
Research screening flowchart

A total of 290 patients were included in this study, with 201 patients in the non-OAC group and 89 in the OAC group. The screening process is shown in Fig. 1. Baseline characteristics of the total cohort, categorized into demographics, comorbidities, laboratory variables, and medications, are detailed in Table 1. Detailed baseline characteristics of patients receiving DOAC therapy, stratified by dosing appropriateness (appropriately dosed vs. inappropriately underdosed), are provided in *Supplementary eTable 2*. Among the enrolled patients, patients were very elderly (mean age, 92.52 ± 2.06 years), 70.69% were male patients, and the mean BMI was (23.50 ± 3.04) kg/m². 95.52% of the patients are in a state of malnutrition (CONUT score ≥ 2). 28.97% had paroxysmal AF, and the average CHA_2_DS_2_-VASc score and HAS-BLED score were 5.18 ± 1.42 and 2.81 ± 0.74, respectively. Among the enrolled patients, 58.62% were treated in the Department of Cardiovascular, and the remaining 41.38% were treated in the Department of Neurology. Patients receiving parenteral anticoagulant therapy were excluded at the time of enrollment. Among these 290 enrolled nonagenarian patients with atrial fibrillation, 30.69% received oral anticoagulant therapy, while 69.31% did not receive any oral anticoagulant therapy. 94.48% of the patients had comorbidity (with medication use ≥ 3 types). 96.21% and 86.21% had polypharmacy (with medication use ≥ 5 types) and hyperpolypharmacy (≥ 10 types).

**Table 1:** Regression analysis of factors associated with anticoagulant therapy use or non-use in patients

	Non-OAC (*n* = 201)	OAC (*n* = 89)	*p* value
Demographics			
Male, n (%)		205 (70.69)	
Age, year (IQR)	92.00 (91.00–94.00)	92.00 (91.00–93.00)	*0.134*
BMI, kg/m² (IQR)	23.87 (22.10-24.91)	23.57 (20.57–25.75)	*0.950*
SBP, mmHg (IQR)	145.00 (126.00-158.00)	153.00 (133.00-159.00)	*0.083*
DBP, mmHg (IQR)	73.00 (65.00–79.00)	77.00 (67.00–82.00)	*0.246*
HR, mmHg (IQR)	75.00 (70.00–80.00)	74.00 (69.00–82.00)	*0.613*
Smoking status, n (%)	23 (11.44)	14 (15.73)	*0.313*
Drinking status, n (%)	35 (17.41)	17 (19.10)	*0.730*
Bedridden status, n (%)	87 (43.28)	5 (5.62)	*< 0.001*
CONUT score(IQR)	4.00 (3.00–5.00)	4.00 (3.00–5.00)	*0.640*
Normal, 0–1	7 (3.48)	6 (6.74)	*0.099*
Mild, 2–4	135 (67.16)	58 (65.17)	*0.304*
Moderate, 5–8	59 (29.35)	25 (28.09)	*0.799*
Severe, 9–12	0	0	*-*
Malnutrition, n (%)	194 (96.52)	83 (93.26)	*0.133*
LOS, day (IQR)	17.00 (7.00–24.00)	8.00 (6.00–15.00)	*< 0.001*
**Anticoagulation Indications**			
Atrial Flutter, n (%)	12 (5.97)	2 (2.25)	*0.240*
Paroxysmal AF, n (%)	46 (22.89)	38 (42.70)	*0.001*
Non-paroxysmal AF, n (%)	44 (21.89)	35 (39.33)	*0.002*
AFNOS, n (%)	99 (49.25)	14 (15.73)	*< 0.001*
**Risk stratification scores**			
CHA_2_DS_2_-VASc (IQR)	4.00 (4.00–5.00)	4.00 (3.00–5.00)	*0.517*
HAS-BLED (IQR)	3.00 (3.00–3.00)	3.00 (2.00–3.00)	*< 0.001*
**Admission department**			
Department of Cardiology, n (%)	91 (45.27)	79 (88.76)	*< 0.001*
Department of Neurology, n (%)	110 (54.73)	10 (11.24)	*< 0.001*
**Types of DOAC**			
Rivaroxaban, n (%)	-	29 (32.58)	*-*
Edoxaban, n (%)	-	37 (41.57)	*-*
Dabigatran, n (%)	-	7 (7.87)	*-*
**Anticoagulant dose**			
DOAC recommended dose, n (%)	-	27 (30.34)	*-*
DOAC low dose, n (%)	-	46 (51.69)	*-*
Warfarin dose, mg/d (IQR)	-	1.875 (1.500-1.875)	*-*
**Comorbidities**			
Comorbidity, ≥ 3	193 (96.02)	81 (91.01)	*0.133*
Hypertension, n (%)	174 (86.57)	71 (79.78)	*0.141*
DM, n (%)	68 (33.83)	18 (20.23)	*0.019*
Hyperlipemia, n (%)	134 (66.67)	44 (49.44)	*0.005*
CKD, n (%)	94 (46.77)	47 (52.81)	*0.342*
COPD, n (%)	15 (7.46)	10 (11.24)	*0.291*
HF, n (%)	31 (15.42)	38 (42.70)	*< 0.001*
CHD, n (%)	85 (42.29)	45 (50.56)	*0.191*
Previous PCI, n (%)	26 (12.94)	23 (25.84)	*0.007*
Previous CABG, n (%)	0	1 (1.24)	*0.307*
VHD, n (%)	14 (6.97)	20 (22.47)	*< 0.001*
Cardiomyopathy, n (%)	35 (17.41)	14 (15.73)	*0.724*
Previous RFA, n (%)	9 (4.48)	9 (10.11)	*0.067*
Previous CIED, n (%)	116 (57.71)	25 (28.09)	*< 0.001*
Ischemic stroke, n (%)	119 (59.20)	30 (33.71)	*< 0.001*
PAD, n (%)	34 (16.92)	13 (14.61)	*0.623*
Thyroid disease, n (%)	21 (10.45)	10 (11.24)	*0.841*
Hyperuricemia, n (%)	66 (32.84)	13 (14.61)	*0.001*
OSAHS, n (%)	3 (1.49)	2 (2.25)	*0.644*
Liver dysfunction, n (%)	11 (5.47)	6 (6.74)	*0.671*
Cancer, n (%)	70 (34.83)	14 (15.73)	*0.001*
History of bleeding, n (%)	93 (46.27)	9 (10.11)	*< 0.001*
Previous ICH, n (%)	2 (1.00)	0	*1.000*
History of GIB, n (%)	4 (1.99)	2 (2.25)	*1.000*
Previous VTE, n (%)	77 (38.31)	6 (6.74)	*< 0.001*
**Laboratory variables**			
Hemoglobin, g/dL (IQR)	11.40 (10.20–12.10)	11.40 (10.26–12.80)	*0.752*
Platelets, ×10^9^/L (IQR)	174.00 (135.00-194.00)	181.00 (159.00-217.00)	*0.050*
Albumin, g/L (IQR)	35.00 (33.00–37.00)	36.00 (33.00–38.00)	*0.288*
TC, mmol/L (IQR)	3.48 (3.31–3.69)	3.46 (2.88–3.83)	*0.764*
LDL-C, mmol/L (IQR)	1.93 (1.76–2.10)	1.85 (1.47–2.11)	*0.533*
ALT, U/L (IQR)	12.00 (8.00–19.00)	15.00 (11.00–21.00)	*0.991*
AST, U/L (IQR)	19.00 (15.00–30.00)	20.00 (16.00–25.00)	*0.482*
CK, U/L (IQR)	61.00 (38.00–83.00)	62.00 (49.00–83.00)	*0.228*
Scr, mg/dL (IQR)	87.00 (64.00-111.00)	87.00 (75.00-106.00)	*0.656*
Serum Uric Acid, µmol/L (IQR)	370.00 (285.00-451.00)	342.00 (294.00-441.00)	*0.493*
eGFR, ml/min/1.73 m² (IQR)	64.49 (48.04–78.70)	60.45 (45.21–73.62)	*0.082*
**Medications**			
Polypharmacy, ≥ 5	198 (98.51)	81 (91.01)	*0.477*
Hyperpolypharmacy, ≥ 10	177 (88.06)	73 (82.02)	*0.715*
Antiplatelet, n (%)	119 (59.20)	20 (22.47)	*< 0.001*
PPI, n (%)	148 (73.63)	35 (39.33)	*< 0.001*
Amiodarone, n (%)	10 (4.98)	5 (5.62)	*0.781*
Digoxin, n (%)	45 (22.39)	18 (20.23)	*0.680*
ACEI/ARB, n (%)	33 (16.42)	34 (38.20)	*< 0.001*
β-blocker, n (%)	65 (32.34)	51 (57.30)	*< 0.001*
Diltiazem/verapamil, n (%)	37 (18.41)	4 (4.50)	*0.002*
CCB, n (%)	69 (34.33)	33 (37.08)	*0.651*
ARNI, n (%)	15 (7.46)	19 (21.35)	*0.001*
Diuretic, n (%)	66 (32.84)	42 (47.19)	*0.020*
Statin, n (%)	137 (68.16)	55 (61.80)	*0.291*

*OAC* oral anticoagulant, *DOAC* direct oral anticoagulant, *IQR* interquartile range, *BMI* body mass index, *SBP* systolic blood pressure, *DBP* diastolic blood pressure, *HR* heart rate, *LOS* length of stay, *AF* atrial fibrillation, *AFNOS* atrial fibrillation not otherwise specified, *DM* diabetes mellitus, *CKD* chronic kidney disease, *COPD* chronic obstructive pulmonary disease, *HF* heart failure, *CHD* coronary heart disease, *PCI* percutaneous coronary intervention, *CABG* coronary artery bypass grafting, *VHD* valvular heart disease, *RFA* radiofrequency ablation, *CIED* cardiac implantable electronic device, *PAD* peripheral artery disease, *OSAHS* obstructive sleep apnea-hypopnea syndrome, *ICH* intracerebral hemorrhage, *GIB* gastrointestinal bleeding, *VTE* venous thrombosis, *TC* total cholesterol, *LDL-C* low-density lipoprotein cholesterol, *ALT* alanine transaminase, *AST* aspartate transaminase, *CK* creatine kinase, *PPI* proton pump inhibitor, *ACEI* angiotensin-converting enzyme inhibitor, *ARB* angiotensin Ⅱ receptor blocker, *CCB* calcium channel blocker, *ARNI* angiotensin receptor-neprilysin inhibitor

It can be seen from the univariate analysis that compared with the non-OAC group, the OAC group had a lower proportion of bedridden patients (*p* < 0.001) and a shorter length of hospital stay (*p* < 0.001). However, no statistically significant differences were observed between the two groups in terms of gender, age, BMI, blood pressure, heart rate, smoking and drinking status, or nutritional status. The OAC group had a lower HAS-BLED score (*p* < 0.001). Patients hospitalized in the Department of Cardiology tend to receive more aggressive oral anticoagulant therapy, while the proportion of patients receiving oral anticoagulant therapy is lower among those hospitalized in the Department of Neurology (*p* < 0.001) (Table 2).

**Table 2:** Primary and secondary efficacy and safety end points of patients with or without oral anticoagulant therapy

	Non-OAC (*n* = 201)	OAC (*n* = 89)	OR (95%CI)	*p* value
	no. of patients with event (%)*			
**Primary efficacy end points**†				
Stroke or systemic embolism	35 (17.41)	6 (6.74)	0.343 (0.139–0.848)	*0.020*
Stroke	18 (8.96)	3 (3.37)	0.355 (0.102–1.236)	*0.104*
Systemic embolism	17 (8.46)	3 (3.37)	0.378 (0.108–1.323)	*0.128*
**Secondary efficacy end points**†				
Stroke, systemic embolism, or death from any cause	66 (32.84)	16 (17.98)	0.448 (0.242–0.830)	*0.011*
Net clinical benefit‡	66 (32.84)	18 (20.22)	0.519 (0.286–0.940)	*0.031*
Death from any cause	20 (9.95)	3 (3.37)	0.316 (0.091–1.091)	*0.068*
**Primary safety end point**†				
Major bleeding	1 (0.50)	2 (2.25)	4.598 (0.411–51.376)	*0.215*
**Secondary safety end points**†				
Major or clinical relevant nonmajor bleeding	21 (10.45)	14 (15.73)	1.600 (0.773–3.313)	*0.206*
Clinical relevant nonmajor bleeding	20 (9.95)	13 (14.61)	1.548 (0.733–3.270)	*0.252*
Minor bleeding	53 (26.37)	32 (35.96)	1.156 (0.918–2.676)	*0.099*
All bleeding events	73 (36.32)	44 (49.44)	1.714 (1.034–2.842)	*0.037*

* The % here refers to the percentage of in-hospital events in the group accounting for the total number of cases in the group

† Both safety and efficacy end points were assessed during the patients’ hospitalization period

‡ Net clinical benefit was the composite of stroke, systemic embolism, major bleeding, or death from any cause

### Multivariable predictors of OAC prescription

The multivariable logistic regression model identified specific independent factors driving the clinical decision to prescribe OAC therapy (Table 3). Patients with high-thrombotic-risk conditions were significantly favored for OAC use; specifically, a history of ischemic stroke (OR = 2.653, 95% CI: 1.190–5.917, *P* = 0.017) and heart failure (OR = 2.083, 95% CI: 1.065–4.082, *P* = 0.032) were independently associated with an increased likelihood of receiving OACs. Conversely, severe frailty and concurrent bleeding risks strongly deterred OAC prescription. Patients with a bedridden status (OR = 0.134, 95% CI: 0.040–0.450, *P* = 0.001) and those receiving concomitant antiplatelet therapy (OR = 0.203, 95% CI: 0.103–0.397, *P* < 0.001) were substantially less likely to be managed with OACs.

**Table 3:** Multivariable logistic regression model predicting the use of oral anticoagulants (OAC)

Variables	OR	95% CI	*P* value
Admission department (Cardiology vs. Neurology)	2.653	0.942–7.519	*0.065*
Ischemic stroke	2.653	1.190–5.917	*0.017*
History of bleeding	0.540	0.219–1.330	*0.180*
Heart failure	2.083	1.065–4.082	*0.032*
Cancer	0.462	0.211–1.014	*0.054*
Concomitant antiplatelet use	0.203	0.103–0.397	*< 0.001*
Bedridden status	0.134	0.040–0.450	*0.001*

### Temporal trends of OAC therapy

**Fig. 2:**
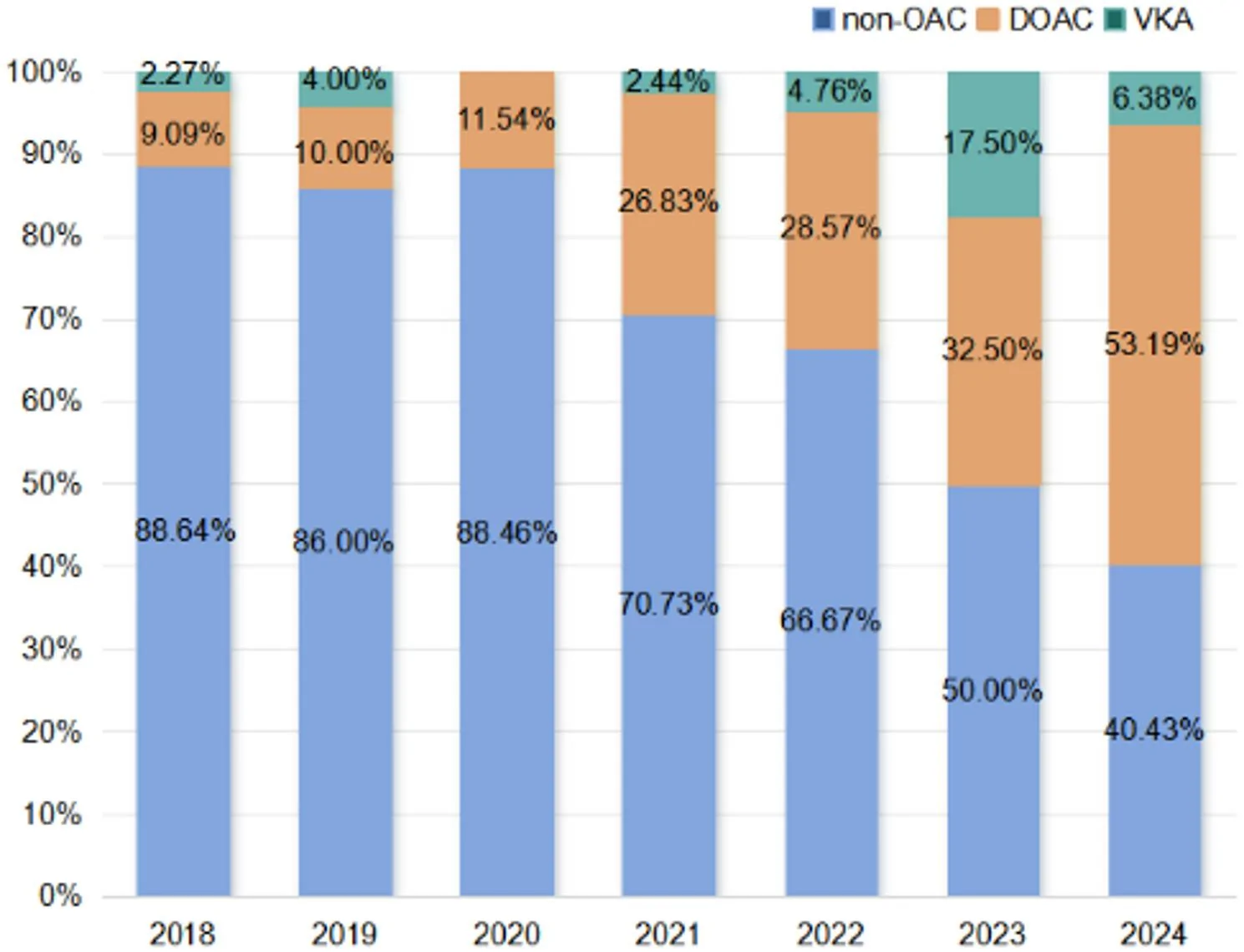
Temporal trends in the prescription of anticoagulant therapy in AF patients aged ≥ 90 years

Figure 2 shows the annual changing trend of anticoagulant therapy in this population. From 2018 to 2024, the proportion of elderly nonagenarian AF patients receiving OAC has been increasing, and it can be seen that an increasing number of nonagenarians tend to receive DOACs for such therapy.

### Appropriateness of prescribed DOAC dose levels

A total of 73 patients received DOAC therapy, with 27 patients (36.99%) in recommended dose group and 46 (63.01%) in the inappropriately underdosing group (*Supplementary eTable 2*). The mean age was (92.15 ± 2.06) years old. 64.38% of this population were male. 91.78% or the patients were categorized as malnutrition. 90.41% of the patients had comorbidity (with medication use ≥ 3 types), and 94.52% had polypharmacy (with medication use ≥ 5 types). *eTable 2* in *Supplementary* shows the differences in various clinical parameters between the two groups. Patients receiving inappropriately underdose DOAC were consistent with the overall population in terms of demographics, comorbidity, and polypharmacy. Among patients receiving inappropriately underdose DOAC group, rivaroxaban (56.52% of the total inappropriately underdosing group) was more frequently preferred. In the recommended dose DOAC group, edoxaban (81.48% of the total recommended dose group) had the highest proportion. In terms of concomitant medication use, the inappropriately underdosing group had a higher rate of antiplatelet drug use compared with the recommended-dose group (*p* = 0.002).

### Efficacy end points

A total of 41 in-hospital events of stroke/SE (the primary end point) were reported. 6 patients (6.74%) in the OAC group and 31 patients (15.42%) in the non-OAC group had stroke/SE (OR, 0.396; 95% CI, 0.159 to 0.987, *p* = 0.047) (Table 4).

There were 3 (3.37%) deaths from any cause in the OAC group and 20 (9.95%) in the non-OAC group (OR, 0.316; 95% CI, 0.091 to 1.091, *p* = 0.068) (Table 4).

The rate of secondary efficacy end point (the composite of stroke, systemic embolism, or death from any cause) was 17.98% in the OAC group and 30.85% in the non-OAC group (OR, 0.491; 95% CI, 0.265 to 0.912, *p* = 0.024). Futhermore, compared to the non-OAC group, the OAC group demonstrated a numerically lower incidence in the composite of net clinical benefit (stroke, systemic embolism, major bleeding, or death from any cause) (20.22% vs. 30.85%), although this difference did not reach statistical significance (Table 4; OR, 0.569; 95% CI, 0.313 to 1.033, *p* = 0.064).

### Safety end points

In the safety population, there were 2 events of major bleeding (2.25%) in the OAC group and 1 (0.50%) in the non-OAC group (OR, 4.598; 95% CI, 0.411 to 51.376; *p* = 0.215) (Table 4). In the secondary endpoint events, the number of total bleeding events was 44 cases (49.44%) in the OAC group and 73 cases (36.32%) in the non-OAC group. Regarding the incidence of other secondary safety endpoints—including the composite endpoint of major bleeding or clinically relevant nonmajor bleeding, clinically relevant nonmajor bleeding, and minor bleeding events—no statistically significant difference was observed compared with the non-OAC group (Table 4).

**Table 4:** Primary and secondary efficacy and safety end points of patients with or without oral anticoagulant therapy

	Non-OAC (*n* = 201)	OAC (*n* = 89)	OR (95%CI)	*P* value
	no. of patients with event (%)*			
**Primary efficacy end points**†				
Stroke or systemic embolism	31 (15.42)	6 (6.74)	0.396 (0.159–0.987)	*0.047*
Stroke	14 (6.97)	3 (3.37)	0.466 (0.130–1.664)	*0.240*
Systemic embolism	17 (8.46)	3 (3.37)	0.378 (0.108–1.323)	*0.128*
**Secondary efficacy end points**†				
Stroke, systemic embolism, or death from any cause	62 (30.85)	16 (17.98)	0.491 (0.265–0.912)	*0.024*
Net clinical benefit‡	62 (30.85)	18 (20.22)	0.569 (0.313–1.033)	*0.064*
Death from any cause	20 (9.95)	3 (3.37)	0.316 (0.091–1.091)	*0.068*
**Primary safety end point**†				
Major bleeding	1 (0.50)	2 (2.25)	4.598 (0.411–51.376)	*0.215*
**Secondary safety end points**†				
Major or clinical relevant nonmajor bleeding	21 (10.45)	14 (15.73)	1.600 (0.773–3.313)	*0.206*
Clinical relevant nonmajor bleeding	20 (9.95)	13 (14.61)	1.548 (0.733–3.270)	*0.252*
Minor bleeding	53 (26.37)	32 (35.96)	1.156 (0.918–2.676)	*0.099*
All bleeding events	73 (36.32)	44 (49.44)	1.714 (1.034–2.842)	*0.037*

* The % here refers to the percentage of in-hospital events in the group accounting for the total number of cases in the group

† Both safety and efficacy end points were assessed during the patients’ hospitalization period

‡ Net clinical benefit was the composite of stroke, systemic embolism, major bleeding, or death from any cause

## Discussion

Until now, real-world evidence on the effectiveness and safety of anticoagulation therapy has been particularly scarce for nonagenarians (≥ 90 years) with AF. In the present study, we exclusively analyze the usage pattern, predictors, and in-hospital outcomes in this group.

The main finding of this study are: (1) OAC was prescribed to 30.69% of nonagenarian AF patients, with a consistent annual increase from 2018 to 2024, and DOACs becoming the preferred choice. (2) Key positive predictors of OAC prescription included a history of ischemic stroke and heart failure, while severe frailty (bedridden status) and concurrent bleeding risks (antiplatelet use) strongly deterred OAC prescription. (3) DOAC accounted for 82.02% of OAC prescriptions; however, 63.01% were inappropriately underdosed, with rivaroxaban being the most common agent in this underdosed group and edoxaban dominating the appropriately dosed group. (4) OAC use was significantly associated with a lower risk of stroke/SE, as well as better outcomes in composite efficacy endpoints (stroke/SE/death from any cause) and net clinical benefit (stroke/SE/major bleeding/death from any cause), while demonstrating a favorable numerical trend in net clinical benefit. (5) Major bleeding occurred in 2.25% of the OAC group vs. 0.50% of the non-OAC group without statistical significance. Total bleeding events were higher in the OAC group, but with no significant disparities in secondary safety endpoints (CRNMB, minor bleeding).

### OAC utilization rate and temporal trends in nonagenarian AF patients

Our study found that overall OAC utilization among nonagenarians remains relatively low, with DOACs dominating the prescription and a steady annual increase in OAC use from 2018 to 2024. This utilization rate is lower than that reported in other real-world studies focusing on elderly AF populations. For example, the AVOPA study reported a 67.3% OAC prescription rate in patients > 85 years [13], and the ANAFIE registry showed an 89.0% OAC use rate in high-bleeding-risk patients ≥ 80 years [14]. The relatively low rate in our cohort may be attributed to the ultra-advanced age (≥ 90 years) and higher burden of frailty-related factors, including widespread malnutrition, polypharmacy, and high comorbidity, which increase clinician hesitancy toward anticoagulation.

Beyond objective clinical vulnerabilities, this persistent between guideline recommendations and real-world clinical practice stems from a complex interplay of physician-, patient-, and family-level apprehensions. In extreme old age, clinicians and family members frequently exhibit an asymmetric perception of risk—disproportionately amplifying the fear of fatal bleeding complications (such as intracranial hemorrhage from accidental falls) while discounting the long-term benefits of stroke prevention. Furthermore, challenges associated with assessing cognitive impairment, managing complex polypharmacy regimens, and monitoring shifting renal function in nonagenarians create a pervasive clinical inertia, ultimately leading to the under-prescription of OACs in this vulnerable demographic.

Consistent with Zhang et al. (2025)’s findings in ≥ 80-year-old AF inpatients, DOACs have become the preferred OAC in our nonagenarian cohort [15], replacing VKAs due to their possible favorable pharmacokinetic profiles (fixed dosing, no routine monitoring) and lower intracranial bleeding risk [16]. The gradual increase in OAC prescription rates also reflects the impact of updated clinical guidelines that emphasize individualized risk-benefit assessment over age-based exclusion from anticoagulation [7]. However, the persistent underutilization suggests that barriers such as perceived bleeding risk in ultra-elderly patients remain unaddressed, highlighting the need for further education on DOAC safety in this population.

### Predictors of OAC prescription

Multivariate logistic regression identified several key predictors of OAC use, highlighting a risk-treatment paradox in clinical decision-making. High-thrombotic-risk conditions, specifically a history of ischemic stroke and heart failure, were robust positive predictors. This indicates that clinicians appropriately prioritize secondary prevention and manage high-burden cardiovascular comorbidities with more aggressive anticoagulation.

Conversely, the strong negative association between bedridden status and OAC use aligns with AVOPA study [13], which reported that frailty and functional decline are linked to lower anticoagulation rates due to concerns about falls, impaired hemostasis, and reduced life expectancy. Additionally, the inverse relationship between concomitant antiplatelet use and OAC prescription mirrors clinical practice patterns observed in the ANAFIE registry [14], where dual anti-thrombotic therapy was avoided in high-bleeding-risk patients. However, this cautious approach may overlook potential benefits in select subgroups (e.g., post-PCI patients). highlighting the need for standardized dual anti-thrombotic algorithms for ultra-elderly AF patients.

Recent real-world data underscore the impact of frailty and physical performance on anticoagulation management in elderly AF populations. Findings from the nationwide Italian START registry showed that individual frailty components—including fall risk, living alone, and lack of social support—frequently hinder DOAC prescription in octogenarians and nonagenarians [17]. Nonetheless, DOAC use was associated with lower mortality and cardiovascular risks compared to VKAs across all age and comorbidity strata, without increasing major bleeding [17]. Additionally, reduced physical performance, evaluated by the Short Physical Performance Battery (SPPB), has been identified as an independent predictor of adverse cardiovascular outcomes and mortality in hospitalized elderly AF patients on DOACs [18].

Importantly, the decision to initiate OAC in nonagenarians should not be dictated by chronological age alone, but rather framed within the broader concept of clinical complexity. As our cohort demonstrates, this population is characterized by a high burden of multidimensional vulnerabilities, including profound malnutrition, polypharmacy, and extensive comorbidities. Some extremely elderly patients may be suitable candidates for OAC, while chronologically younger, yet highly frail patients might not. Therefore, future clinical decision-making must transition toward a comprehensive multidimensional assessment—incorporating frailty, cognitive status, and bleeding risk domains—to truly optimize net clinical benefit, aligning with contemporary explorations of complexity domains across geriatric populations [17–19].

### Appropriateness of DOAC dosing in nonagenarians

Among DOAC users, 63.01% received inappropriately underdose, with rivaroxaban being the most common inappropriately underdosing agent (56.52%) and edoxaban dominating the recommended-dose group (81.48%). This inappropriately underdose prevalence is higher than that reported in Zhang et al. (2025) (45.6% low-dose)^14^ and Cavillon Decaestecker et al. (2021) (34% low-dose) [20], which may be explained by the ultra-advanced age (≥ 90 years) of our cohort and high prevalence of dose-reduction criteria: eGFR median = 56.53 ml/min/1.73 m², HAS-BLED median = 3, malnutrition (91.78%), and polypharmacy (94.52%) (*eTable 2*). These factors align with NMPA guidelines recommending reduced-dose DOACs for elderly patients with renal impairment or high bleeding risk.

The dosing pattern observed in our study is consistent with the ELDERCARE-AF trial [7], where low-dose edoxaban (15 mg) was effective and safe in ≥ 80-year-old high-bleeding-risk patients. However, the appropriateness of low-dose DOACs in nonagenarians remains debated: a post hoc analysis of the ENGAGE AF-TIMI 48 trial showed that reduced-dose edoxaban (30 mg) in ≥ 80-year-olds reduced major bleeding without increasing ischemic events [21], while the GARFIELD-AF registry reported that underdosing was associated with higher all-cause mortality in younger AF patients [22]. A striking correlate was the significantly higher antiplatelet co-prescription in low-dose users (34.78% vs. 3.70%, *p* = 0.002), reflecting clinician caution in dual anti-thrombotic scenarios—consistent with Zhang et al. (2025)’s observation that polypharmacy and high bleeding risk drive low-dose DOAC use in ≥ 80-year-olds.^14^ Given the lack of nonagenarian-specific dosing guidelines, these eTable 2-derived patterns underscore the need for dedicated trials to validate low vs. recommended doses in this cohort.

### Efficacy of OAC in nonagenarian AF patients

A key finding of our study is that OAC use was significantly associated with a lower incidence of in-hospital stroke/SE as well as more favorable outcomes regarding the composite efficacy endpoints (stroke/SE/death from any cause). Furthermore, the OAC group demonstrated a numerically lower trend in net clinical benefit (stroke/SE/major bleeding/death from any cause). This aligns with the ELDERCARE-AF trial, which low-dose edoxaban (15 mg) reduced stroke/SE by 64% (HR = 0.34) in ≥ 80-year-old high-bleeding-risk patients compared to placebo [7]. Notably, our study extends this evidence to an even older subgroup (≥ 90 years), a population historically excluded from pivotal DOAC trials (e.g., ENGAGE AF-TIMI 48 included only 17% of patients ≥ 80 years) [3].

Our results also confirm the findings of a nationwide Korean cohort study, which reported that DOACs reduced ischemic stroke risk (HR = 0.81) and improved net clinical benefit in ≥ 80-year-old high-bleeding-risk AF patients [23]. The consistent efficacy across studies challenges the historical reluctance to prescribe OACs to nonagenarians due to perceived frailty, emphasizing that stroke risk reduction outweighs potential bleeding concerns in this population. The net clinical benefit observed in our study further supports the “risk-benefit paradigm” advocated by current guidelines [24], underscoring that age alone should not be a contraindication to OAC therapy.

### Safety PROFILE of OAC in nonagenarian AF patients

Despite a higher total bleeding rate in the OAC group (49.44% vs. 36.32%, *p* = 0.037), major bleeding (2.25% vs. 0.50%, *p* = 0.215) and secondary safety endpoints (CRNMB, minor bleeding) showed no statistically significant differences between OAC and non-OAC groups. This acceptable safety profile is consistent with DOAC data in elderly populations: the COMBINE-AF subanalysis demonstrated that frail ≥ 75-year-old patients switching to standard-dose DOACs had similar major bleeding rates to warfarin users but significantly lower intracranial bleeding risk [25].

The favorable safety profile of OACs in our nonagenarian cohort can be attributed to two key factors: first, the predominance of DOACs over VKAs, as DOACs are known to have a lower risk of intracranial hemorrhage [15]; second, adherence to dose adjustments based on National Medical Products Administration (NMPA) guidelines (e.g., reduced-dose DOACs for renal impairment or high bleeding risk; *etable*). Notably, our findings are consistent with the ELDERCARE-AF trial,^7^ where low-dose edoxaban did not significantly increase major bleeding compared to placebo (3.3% vs. 1.8%, *p* = 0.09). These results collectively indicate that OACs can be safely prescribed to nonagenarians when individualized risk assessment (e.g., HAS-BLED score, renal function, etc.) is performed.

### Study limitations and future directions

This study has several limitations. First, the retrospective, single-center observational design inherently limits generalizability of our findings. Notably, our cohort had a high male predominance (70.69%), which is atypical for populations aged ≥ 90 years and likely reflects the specific demographic characteristics of patient referrals at our institution. Furthermore, as an observational study, it is highly susceptible to unmeasured confounding and indication bias. Patients selected to receive OACs might represent a “healthier user” subgroup with less severe frailty or fewer unrecorded bleeding risks compared to the non-OAC group, which may partially explain the observed favorable outcomes.

Second, to rigorously address the substantial confounding-by-indication associated with pre-admission strokes, we explicitly restricted our primary efficacy endpoint to incident strokes occurring strictly during hospitalization. While this adjustment accurately reflects the in-hospital treatment effect and minimizes selection bias, our findings are consequently confined to the short-term acute care setting. Additionally, by exclusively enrolling hospitalized patients, we inherently selected a subgroup with higher acute acuity, which may overestimate risks compared to community-dwelling nonagenarians.

Third, our study was underpowered, particularly regarding safety outcomes. With a small OAC cohort (*n* = 89) and an extremely low number of total major bleeding events, the wide confidence intervals indicate that cautious interpretation is necessary. However, to address the limited sample size in our multivariable regression analysis, we adopted a rigorously parsimonious approach. By selectively including only the most critical clinical confounders to strictly adhere to the events-per-variable (EPV) ≥ 10 principle, we successfully mitigated the risk of model overfitting, thereby ensuring the statistical robustness of the identified independent predictors for OAC prescription.

Fourth, the evaluation was restricted to short-term in-hospital outcomes (median length of stay: 14 days). In such an advanced-age cohort, the high all-cause mortality introduces a significant competing risk. Due to the short and acute timeframe, we utilized multivariate logistic regression rather than formal time-to-event or competing risk models (e.g., Fine-Gray subdistribution hazard models). While pragmatic for this specific acute timeframe, the lack of long-term follow-up remains a limitation, and the differential length of stay between groups may have introduced unmeasured time-related biases.

Fifth, for the small subset of patients treated with Vitamin K antagonists (*n* = 16), data regarding precise INR control and time in therapeutic range (TTR) were not systematically available, limiting our ability to assess the quality of warfarin anticoagulation.

Future research should prioritize large-scale, multi-center prospective studies with long-term follow-up to validate these findings and adequately power the evaluation of safety outcomes. Furthermore, time-to-event analyses incorporating competing risks, randomized trials clarifying optimal DOAC dosing (low vs. recommended), and the development of standardized algorithms incorporating frailty and polypharmacy are urgently needed to optimize net clinical benefit in this highly complex, ultra-elderly population.

## Conclusions

In conclusion, our study provides real-world evidence for anticoagulation therapy in patients with AF aged ≥ 90 years. Although the overall utilization rate of OACs remains relatively low in this group, it has shown a steady annual increase, with DOAC emerging as the predominant choice. Critically, OAC therapy is significantly associated with a lower risk of in-hospital stroke and yields favorable net clinical benefit, highlighting the potential value of the judicious use of OACs in this population rather than age-based exclusion. Furthermore, our data regarding the safety, efficacy, dosing patterns, and prescription predictors of OACs add supplementary real-world insights for this understudied population. These findings validate the importance of individualized risk-benefit assessment and provide practical data to help refine clinical management for ultra-elderly AF patients.

## Supplementary Information

Supplementary Material 1.

## Data Availability

The datasets generated and analyzed during the current study are not publicly available due to institutional confidentiality requirements and the need to protect patients’ individual privacy. However, de-identified data are available from the corresponding author on reasonable request.

## Abbreviations

ACEI: Angiotensin-converting enzyme inhibitor
AF: Atrial fibrillation
AFNOS: Atrial fibrillation not otherwise specified
ARB: Angiotensin II receptor blocker
ARNI: Angiotensin receptor-neprilysin inhibitor
BMI: Body mass index
CI: Confidence interval
CIED: Cardiac implantable electronic device
CONUT: Controlling Nutritional Status
CrCl: Creatinine clearance
DBP: Diastolic blood pressure
DM: Diabetes mellitus
DOAC: Direct oral anticoagulant
eGFR: Estimated glomerular filtration rate
ESC: European Society of Cardiology
HF: Heart failure
HIS: Hospital information system
HR: Heart rate
ICH: Intracranial hemorrhage
IQR: Interquartile range
ISTH: International Society on Thrombosis and Haemostasis
LOS: Length of stay (or length of hospital stay)
NMPA: National Medical Products Administration
OAC: Oral anticoagulant
OR: Odds ratio
PCI: Percutaneous coronary intervention
PPI: Proton pump inhibitor
RRR: Relative risk reduction
SBP: Systolic blood pressure
SE: Systemic embolism
SPPB: Short Physical Performance Battery
VHD: Valvular heart disease
VKA: Vitamin K antagonist
VTE: Venous thrombosis
