# Supplementary material for: Oral anticoagulation therapy in nonagenarian patients with atrial fibrillation: usage patterns, predictors, and in-hospital outcomes from a single-center retrospective study
Source: BMC Geriatr. 2026 Aug 8;26:1396. doi: 10.1186/s12877-026-08098-y (PMC13750039; doi:10.1186/s12877-026-08098-y)
Supplement: Supplementary file 1 [file 12877_2026_8098_MOESM1_ESM.docx]

**eTable 1** Approved dosing regimens for the NOACs package inserts for AF in the Mainland China

| NOACs | Regulations from the NMPA (Mainland China) |
| --- | --- |
| Dabigatran | - Full dose: 150 mg twice daily - 110 mg twice daily if: (1) age ≥ 80 years; (2) concomitant verapamil - Daily dose of 300 mg or 220 mg according to an individual evaluation of the thromboembolic risk and bleeding risk: (1) 75 ≤ age < 80 years; (2) moderate renal impairment (30 ≤ CrCl < 50 ml/min); (3) gastritis, esophagitis, or gastroesophageal reflux; (4) other increased bleeding risk* |
| Rivaroxaban | - Full dose: 20 mg once daily with food when CrCl ≥ 50 mL/min - 15 mg once daily with food, if: 15 ≤ CrCl < 50 mL/min; (2) age > 75 years old |
| Edoxaban | - Full dose: 60 mg once daily - 30 mg once daily with one or more of the following clinical factors: (1) 15 ≤ CrCl < 50 mL/min; (2) body weight ≤ 60 kg; (3) concomitant use of the following P-gp inhibitors including ciclosporin, erythromycin, dronedarone, or ketoconazole |

* Other increased bleeding risks include: Strong P-gp inhibitors; mild to moderate P-gp inhibitor co-medication (e.g., quinidine, verapamil, ticagrelor, and amiodarone); low body weight (< 50 kg); acetylsalicylic acid (ASA) and other platelet aggregation inhibitors, e.g., clopidogrel; selective serotonin norepinephrine re-uptake inhibitors (SNRIs), selective serotonin re-uptake inhibitors (SSRIs), and non-steroidal anti-inflammatory drugs (NSAID); other medicinal products that may impair hemostasis; functional platelet defects or thrombocytopenia; major trauma and recent biopsy; and bacterial endocarditis.

**eTable 2** Baseline characteristics of patients receiving DOAC therapy, stratified by dosing appropriateness

|  | | | | **Total DOAC**  **(n = 73)** | **Appropriate dosing**  **(n = 27)** | **Inappropriate underdosing**  **(n = 46)** | ***P* value** |
| --- | --- | --- | --- | --- | --- | --- | --- |
| **Demographics** | | | |  |  |  |  |
|  | Male, n (%) | | | 47 (64.38) | 17 (62.96) | 30 (65.24) | *0.846* |
|  | Age, year (IQR) | | | 91.00 (91.00-93.00) | 92.00 (91.00-93.00) | 91.00 (91.00-92.75) | *0.565* |
|  | BMI, kg/m² (IQR) | | | 23.36 (20.45-25.77) | 24.44 (20.40-26.95) | 23.18 (20.33-25.34) | *0.490* |
|  | SBP, mmHg (IQR) | | | 148.00 (130.00-159.00) | 153.00 (133.50-159.50) | 146.00 (126.25-159.00) | *0.557* |
|  | DBP, mmHg (IQR) | | | 76.00 (65.00-82.00) | 70.00 (65.00-80.00) | 76.50 (67.00-84.00) | *0.481* |
|  | HR, mmHg (IQR) | | | 74.00 (69.00-82.00) | 75.00 (69.00-83.00) | 73.50 (69.00-81.00) | *0.955* |
|  | Smoking status, n (%) | | | 12 (16.44) | 4 (14.85) | 8 (17.39) | *1.000* |
|  | Drinking status, n (%) | | | 17 (23.29) | 6 (22.22) | 11 (23.91) | *0.869* |
|  | Bedridden status, n (%) | | | 5 (6.85) | 1 (3.70) | 4 (8.70) | *0.645* |
|  | LOS, day (IQR) | | | 10.00 (6.00-18.00) | 10.00 (6.00-20.50) | 10.50 (7.00-17.50) | *0.967* |
|  | CHA_2_DS_2_-VASc (IQR) | | | 5.00 (3.00-5.00) | 5.00 (4.00-5.50) | 4.50 (3.00-5.00) | *0.458* |
|  | HAS-BLED (IQR) | | | 3.00 (2.00-3.00) | 2.00 (2.00-3.00) | 3.00 (2.00-3.00) | *0.298* |
|  | COUNT (IQR) | | | 4.00 (3.00-5.00) | 4.00 (3.00-4.50) | 4.00 (2.25-5.00) | *0.672* |
|  | Admission department | | |  |  |  |  |
|  |  | Department of Cardiology, n (%) | | 63 (86.30) | 24 (88.89) | 39 (84.78) | *0.735* |
|  |  | Department of Neurology, n (%) | | 10 (13.70) | 3 (11.11) | 7 (15.22) | *0.735* |
| **Comorbidities** | | | |  |  |  |  |
| Comorbidity, ≥ 3 | | | | 66 (90.41) | 26 (96.30) | 40 (86.96) | *0.248* |
|  | Anticoagulation Indications | | |  |  |  |  |
|  |  | | Atrial Flutter, n (%) | 1 (1.37) | 1 (3.70) | 0 | *0.370* |
|  |  | | Paroxysmal AF, n (%) | 32 (43.84) | 10 (37.04) | 22 (47.83) | *0.370* |
|  |  | | Non-paroxysmal AF, n (%) | 28 (38.36) | 12 (44.44) | 16 (34.78) | *0.412* |
|  |  | | AFNOS, n (%) | 12 (16.44) | 4 (14.82) | 8 (17.39) | *1.000* |
|  | Hypertension, n (%) | | | 63 (86.30) | 22 (81.48) | 41 (89.13) | *0.483* |
|  | Diabetes, n (%) | | | 17 (23.29) | 9 (33.33) | 8 (17.39) | *0.120* |
|  | Hyperlipemia, n (%) | | | 33 (45.21) | 11 (40.74) | 22 (47.83) | *0.557* |
|  | Chronic renal disease, n (%) | | | 41 (56.16) | 14 (51.85) | 27 (58.70) | *0.569* |
|  | COPD, n (%) | | | 3 (4.11) | 0 | 3 (6.52) | *0.291* |
|  | Heart Failure, n (%) | | | 29 (39.73) | 11 (40.74) | 18 (39.13) | *0.892* |
|  | Coronary heart disease, n (%) | | | 32 (43.84) | 14 (51.85) | 18 (39.13) | *0.290* |
|  | Previous PCI, n (%) | | | 12 (16.44) | 2 (7.41) | 10 (21.34) | *0.190* |
|  | Previous CABG, n (%) | | | 1 (1.37) | 1 (3.70) | 0 | *0.370* |
|  | Cardiac valvular disease, n (%) | | | 14 (19.18) | 8 (29.63) | 6 (13.04) | *0.082* |
|  | Cardiomyopathy, n (%) | | | 10 (13.70) | 3 (11.11) | 7 (15.22) | *0.735* |
|  | Previous RFA, n (%) | | | 9 (12.33) | 4 (14.82) | 5 (10.87) | *0.718* |
|  | Previous CIED, n (%) | | | 16 (21.92) | 7 (25.93) | 9 (19.57) | *0.526* |
|  | Ischemic stroke, n (%) | | | 28 (38.36) | 8 (29.63) | 20 (43.48) | *0.240* |
|  | PAD, n (%) | | | 13 (17.81) | 7 (25.93) | 6 (13.04) | *0.210* |
|  | Thyroid disease, n (%) | | | 9 (12.33) | 1 (3.70) | 8 (17.39) | *0.141* |
|  | Hyperuricemia, n (%) | | | 11 (15.07) | 3 (11.11) | 8 (17.39) | *0.736* |
|  | OSAHS, n (%) | | | 2 (2.74) | 1 (3.70) | 1 (2.17) | *1.000* |
|  | Liver dysfunction, n (%) | | | 4 (5.48) | 2 (7.41) | 2 (4.35) | *0.623* |
|  | Cancer, n (%) | | | 11 (15.07) | 4 (14.82) | 7 (15.22) | *1.000* |
|  | Previous ICH, n (%) | | | 0 | 0 | 0 | *-* |
|  | History of GIB, n (%) | | | 2 (2.74) | 0 | 2 (4.35) | *0.272* |
|  | History of bleeding, n (%) | | | 8 (10.96) | 1 (3.70) | 7 (15.22) | *0.244* |
|  | Previous VTE, n (%) | | | 6 (8.22) | 2 (7.41) | 4 (8.70) | *1.000* |
| **Laboratory variables** | | | |  |  |  |  |
|  | Hemoglobin, g/dL (IQR) | | | 11.60 (10.50-13.10) | 11.50 (10.50-12.60) | 11.85 (10.73-13.38) | *0.475* |
|  | Platelets, ×10^9^/L (IQR) | | | 174.00 (157.00-223.00) | 190.00 (161.50-232.00) | 167.50 (149.50-217.00) | *0.489* |
|  | Albumin, g/L (IQR) | | | 35.00 (33.00-38.00) | 35.00 (33.00-38.00) | 36.00 (34.00-38.00) | *0.610* |
|  | TC, mmol/L (IQR) | | | 3.51 (2.92-4.03) | 3.59 (2.81-4.21) | 3.50 (2.98-3.91) | *0.459* |
|  | LDL-C, mmol/L (IQR) | | | 1.88 (1.51-2.30) | 1.88 (1.48-2.72) | 1.86 (1.55-2.22) | *0.297* |
|  | ALT, U/L (IQR) | | | 14.00 (11.00-20.00) | 18.00 (10.50-28.00) | 14.00 (11.00-16.00) | *0.536* |
|  | AST, U/L (IQR) | | | 20.00 (15.00-24.00) | 22.00 (16.00-25.00) | 19.50 (15.00-22.00) | *0.833* |
|  | CK, U/L (IQR) | | | 59.00 (42.00-81.00) | 51.00 (32.00-71.50) | 61.50 (54.00-81.00) | *0.114* |
|  | Scr, mg/dL (IQR) | | | 89.00 (73.00-109.00) | 86.00 (70.00-93.00) | 94.00 (74.25-115.50) | *0.212* |
|  | Serum Uric Acid, μmol/L (IQR) | | | 361.00 (294.00-454.00) | 322.00 (286.50-410.50) | 395.50 (294.75-466.50) | *0.363* |
|  | eGFR, ml/min/1.73m² (IQR) | | | 56.53 (45.21-75.58) | 60.80 (49.42-75.66) | 55.96 (40.46-74.91) | *0.329* |
| **Medications** | | | |  |  |  |  |
| Polypharmacy, ≥ 5 | | | | 69 (94.52) | 25 (92.59) | 44 (95.65) | *0.623* |
| Hyperpolypharmacy, ≥ 10 | | | | 62 (84.93) | 23 (85.19) | 39 (84.78) | *1.000* |
|  | Types of DOAC | | |  |  |  |  |
|  |  | Rivaroxaban, n (%) | | 29 (39.73) | 3 (11.11) | 26 (56.52) | *< 0.001* |
|  |  | Edoxaban, n (%) | | 37 (50.69) | 22 (81.48) | 15 (32.61) | *< 0.001* |
|  |  | Dabigatran, n (%) | | 7 (9.59) | 2 (7.41) | 5 (10.87) | *1.000* |
|  | Antiplatelet, n (%) | | | 17 (23.29) | 1 (3.70) | 16 (34.78) | *0.002* |
|  | PPI, n (%) | | | 30 (41.10) | 11 (40.74) | 19 (41.30) | *0.962* |
|  | Amiodarone, n (%) | | | 4 (5.48) | 3 (11.11) | 1 (2.17) | *0.140* |
|  | Digoxin, n (%) | | | 11 (15.07) | 4 (14.82) | 7 (15.22) | *1.000* |
|  | ACEI/ARB, n (%) | | | 30 (41.10) | 11 (40.74) | 19 (41.30) | *0.962* |
|  | β-blocker, n (%) | | | 40 (54.79) | 19 (70.37) | 21 (45.65) | *0.041* |
|  | Diltiazem/verapamil, n (%) | | | 2 (2.74) | 0 | 2 (4.35) | *0.527* |
|  | CCB, n (%) | | | 30 (41.10) | 10 (37.04) | 20 (43.48) | *0.589* |
|  | ARNI, n (%) | | | 18 (24.66) | 6 (22.22) | 12 (26.09) | *0.711* |
|  | Diuretic, n (%) | | | 33 (45.21) | 13 (48.15) | 20 (43.48) | *0.699* |
|  | Statin, n (%) | | | 48 (65.75) | 19 (70.37) | 29 (63.04) | *0.524* |
